# Supplementary figures and images for: Stochastic representation decision theory: How probabilities and values are entangled dual characteristics in cognitive processes
Source: PLoS One. 2020 Dec 14;15(12):e0243661. doi: 10.1371/journal.pone.0243661 (PMC7735623; doi:10.1371/journal.pone.0243661)

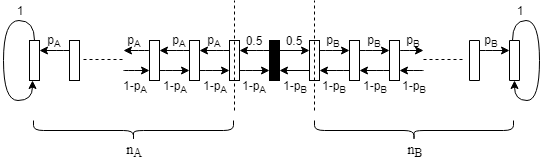

Supplement: S1 Fig — (TIF) [file pone.0243661.s001.tif]

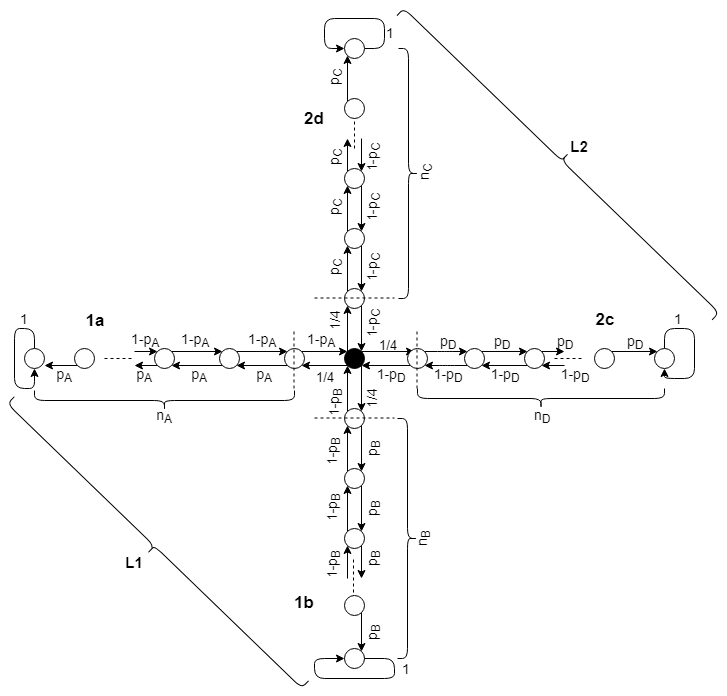

Supplement: S2 Fig — The transition probabilities are different for each segment, in order to correctly represent the different outcome-dependent potentials, while the distance of the absorbing states from the centre is different in each branch, to encode the different lottery probabilities. (TIF) [file pone.0243661.s002.tif]
