# Supplementary material for: Stochastic representation decision theory: How probabilities and values are entangled dual characteristics in cognitive processes
Source: PLoS One. 2020 Dec 14;15(12):e0243661. doi: 10.1371/journal.pone.0243661 (PMC7735623; doi:10.1371/journal.pone.0243661)
Supplement: S1 Appendix — (DOCX) [file pone.0243661.s003.docx]

**Analytical derivation of absorption probabilities**

In this appendix, we show how to derive the absorption probabilities corresponding to subjective probability perception and choice probability, first deriving their exact asymptotic form and then providing their time-dependent approximation. The setup for the subjective probability is a Brownian particle on a line in presence of two absorbing boundaries, while for the decision probability the particle wanders on a “starfish” graph, where each leg represents a lottery outcome (see below). Note that the one-dimensional configuration is just a particular case of the general starfish. For better readability, we recall here the hypothetical binary choice considered

$$L_{1}=\{o_{A},p;o_{B};1-p\} \text{or} L_{2}=\{o_{C},q;o_{D};1-q\}$$

If the DM chooses lottery *L_1_* (resp. *L_2_*), she will receive amount $o_{A}$(resp. $o_{C}$) with probability *p* (resp. *q*), and $o_{B}$(resp. $o_{D}$) with probability *1-p* (resp. *1-q*). The amounts can be negative, corresponding to losses.

**Absorption probabilities in one-dimensional case**

For the derivation of the absorption probabilities of a one-dimensional drifted Brownian motion in presence of two absorbing walls, we follow the procedure outlined by Gardiner [1]. For convenience, we rewrite here our Fokker-Planck equation:

$$\left\{\begin{matrix} \frac{\partial p(x,t)}{\partial t}=-\frac{\partial[-V'(x)p(x,t)]}{\partial x}+\frac{D}{2}\frac{\partial^{2}p(x,t)}{\partial x^{2}} \\ p(a,t)=p(b,t)=0\text{ }\forall t \text{(absorbing boundaries)} \\ p(x,t=0)=\delta(x) \text{(initial condition)} \end{matrix}\right.$$

where $V\left(x\right)$ has the following form

$$V(x)=\left\{\begin{matrix} u(o_{A})x \text{if }x\leq0 \\ -u(o_{B})x \text{if }x>0 \end{matrix}\right.$$

Assume $u\left(o\right)=o$ for ease of notation. Applying the Laplace Transform on Equation we obtain

$$s\tilde{p}\left(x,s\right)-\delta\left(x\right)=\frac{\partial\left[V'\left(x\right)\tilde{p}\left(x,s\right)\right]}{\partial x}+\frac{D}{2}\frac{\partial^{2}\tilde{p}\left(x,s\right)}{\partial x^{2}}$$

where $\tilde{p}\left(x,s\right)=\int_{0}^{\infty} dt\text{ }p(x,t)\text{ }e^{-st}$. Solving Equation separately for $x<0$ and $x>0$ and then imposing the continuity condition $\tilde{p}\left(0^{-},s\right)=\tilde{p}\left(0^{+},s\right)\forall s$we obtain

$$\tilde{p}\left(x,s\right)=\left\{\begin{matrix} A(s)e^{w(o_{A},s)a-\frac{o_{A}x}{D}}\sinh\left(w(o_{A},s)(x-a)\right)\text{, if }x\leq0 \\ A(s)e^{w(o_{A},s)a+\frac{o_{B}x}{D}}\frac{\sinh\left(-w(o_{A},s)a\right)}{\sinh\left(w(o_{B},s)b\right)}\sinh\left(w(o_{B},s)(b-x)\right)\text{, if }x>0 \end{matrix}\right.$$

Where $w\left(o,s\right)\equiv\sqrt{\left(\frac{o}{D}\right)^{2}+\frac{2s}{D}}$ and $A\left(s\right)$ is a constant to determine. To find $A\left(s\right)$ we integrate Equation over the space interval $\left[-\varepsilon,\text{ }\varepsilon\right]$ and then take the limit $\varepsilon\to0$ obtaining

Recalling the expressions for the probability current $J\left(x,t\right)$ and its Laplace transform $\tilde{J}\left(x,s\right)$

$$\begin{matrix} J(x,t)=-V'(x)p(x,t)-\frac{D}{2}\frac{\partial p(x,t)}{\partial x} \\ \tilde{J}(x,s)=\int_{0}^{\infty} dt\text{ }J(x,t)\text{ e}^{-st}=-V'(x)\tilde{p}(x,s)-\frac{D}{2}\frac{\partial\tilde{p}(x,s)}{\partial x} \end{matrix}$$

the probabilities for the particle to be *eventually* absorbed at $x=a<0$ or $x=b>0$, denoted by $P\left(a\right)$ and $P\left(b\right)$- are then given by

$$\begin{matrix} P(a)=-\int_{0}^{\infty} dt\text{ }J(a,t)=-\tilde{J}(a,s=0)=\frac{\frac{o_{A}}{o_{B}}(e^{-\frac{2o_{B}b}{D}}-1)}{\frac{o_{A}}{o_{B}}(e^{-\frac{2o_{B}b}{D}}-1)+(e^{-\frac{2o_{A}\left|a\right|}{D}}-1)} \\ P(b)=1-P(a) \end{matrix}$$

By setting $\left|a\right|=\frac{1}{p}$ and $b=\frac{1}{1-p}$ - where $p$ (resp. $1-p$) is the probability of outcome $o_{A}$ (resp. $o_{B}$) of the lottery $L_{1}$ - we get the desired time-independent subjective probabilities.

The other quantity we are interested in is the probability that the particle is absorbed at, say, $x=a$, conditional on absorption at either wall occurring before time $T$. Denoting it by $P(a|T)$, it is formally given by

$$\begin{matrix} P(a|T)=\frac{\int_{0}^{T} dt\left(-J(a,t)\right)}{\int_{0}^{T} dt\left(-J(a,t)\right)+\int_{0}^{T} dtJ(b,t)} \\ P(b|T)=1-P(a|T) \end{matrix}$$

A closed form solution for Equation is not easily obtainable. However, we can approximate the integrals of the probability current using the following

$$\int_{0}^{T} dtJ(x,t)\cong\int_{0}^{\infty} dtJ(x,t)\text{ e}^{-\frac{t}{T}}=\tilde{J}(x,s=\frac{1}{T})$$

Combining Equations and we obtain

**Absorption probabilities on the “starfish” graph**

The suggested stochastic representation of choice (Fig. 2 in the manuscript) has the advantage of being easily generalizable beyond binary choices while remaining essentially one-dimensional. Calculating directly the absorption probabilities is however quite cumbersome; therefore, we attack the problem by switching to an analogous discrete representation (random walk) both in time and in space, calculating the corresponding absorption probabilities, and finally taking the proper continuous limit.

Let us first apply this procedure to the already solved one-dimensional case, in order to show that the absorption probabilities correspond to the ones obtained in the previous section. Specifically, consider a biased random walk starting at $x=0$ - moving by $\Delta x$ each unit time $\Delta t$ - in presence of two absorbing walls located at $-(n_{A}+1)\Delta x$ and $(n_{B}+1)\Delta x$, as shown in S1 Fig. The transition probabilities are site-dependent

$$p_{right}(x)=\left\{\begin{matrix} \frac{1}{2}\text{ if }x=0 \\ p_{B}\text{ if }0<x<(n_{B}+1)\Delta x \\ (1-p_{A})\text{ if }-(n_{A}+1)\Delta x<x<0 \end{matrix}\right.\text{ }p_{left}(x)=1-p_{right}(x)$$

where $p_{A}+p_{B}\neq1$ in general. Our aim is to calculate the probability for the random walk to be absorbed either at $x=-(n_{A}+1)\Delta x$, denoted by $P_{A}$, or at $x=(n_{B}+1)\Delta x$, denoted by $P_{B}$. This can be easily done expressing, say, $P_{B}$ in terms of absorption probabilities for random walk in presence of uniform bias, whose solution is well known [2]. Specifically, we insert a “fictitious” wall at $x=0$and solve the absorption problem recursively, exploiting the fact that on the right (resp. left) side of $x=0$ we have a simple uniform bias. Consider a simple random walk with right jumping probability$\tilde{p}$, in presence of two absorbing boundaries, the left (resp. right) one located at distance *n* (resp. *m*). Denote with $P_{R}\left(n,m,\tilde{p}\right)$(resp. $P_{L}\left(n,m,\tilde{p}\right)$) its probability to be absorbed by the right (resp. left) wall

$$\begin{matrix} P_{R}\left(n,m,\tilde{p}\right)=\frac{\tilde{p}^{m}\left[\left(1-\tilde{p}\right)^{n}-\tilde{p}^{n}\right]}{\left(1-\tilde{p}\right)^{n+m}-\tilde{p}^{n+m}} \\ P_{L}\left(n,m,\tilde{p}\right)=1-P_{R}\left(n,m,\tilde{p}\right) \end{matrix}$$

Then, $P_{B}$ is found via the following simple recursive relation

$$P_{B}=\frac{1}{2}\left[P_{R}\left(1,n_{B},p_{B}\right)+P_{L}\left(1,n_{B},p_{B}\right)P_{B}\right]+\frac{1}{2}P_{R}\left(n_{A},1,1-p_{A}\right)P_{B}$$

The first term on the r.h.s. of Eq. corresponds to the case where the first step of the particle is to the right (occurring with probability $1/2$); then, the particle either reaches the right wall before coming back to *0* with probability $P_{R}\left(1,n_{B},p_{B}\right)$ or it reaches first *0* with probability $P_{L}\left(1, n_{B}, p_{B}\right)$. If the latter happens, due to Markov property of the walk, the probability to be absorbed by the right wall given that the particle is now in *0* is again $P_{B}$. Similarly, if the first step of the particle is on the left, the probability of being absorbed by the right wall is the probability of reaching first *0*  before the left wall $P_{R}\left(n_{A},1,1-p_{A}\right)$, times the probability of being absorbed by the right wall given that it is in *0*, i.e. $P_{B}$. Solving Eq. for $P_{B}$ gives

$$P_{B}=\frac{P_{R}\left(1,n_{B}, p_{B}\right)}{P_{R}\left(1,n_{B}, p_{B}\right)+P_{L}\left(n_{A},1,1-p_{A}\right)}$$

To retrieve expression , let us recall that a random walk moving by $\Delta x$ to the right (resp. left) with probability $\tilde{p}$ (resp. $1-\tilde{p}$) after $\Delta t$ time converges as $\Delta t\to0$ to a Brownian motion with drift $\mu$ and variance $\sigma^{2}=D$ if the following relations hold:

$$\begin{matrix} \Delta x=\sigma\sqrt{\Delta t} \\ \tilde{p}=\frac{1}{2}[1+\frac{\mu\sqrt{\Delta t}}{\sigma}]n \end{matrix}$$

For our present case, given the potential reported in Eq. and the absorbing boundaries located at $a<0$, $b>0$, we set

$$\begin{matrix} n_{B}=\frac{b}{\Delta x}=\frac{b}{\sigma\sqrt{\Delta t}}, p_{B}=\frac{1}{2}+\frac{u(o_{B})\sqrt{\Delta t}}{2\sigma} \\ n_{A}=\frac{\left|a\right|}{\Delta x}=\frac{\left|a\right|}{\sigma\sqrt{\Delta t}}, p_{A}=\frac{1}{2}+\frac{u(o_{A})\sqrt{\Delta t}}{2\sigma} \end{matrix}$$

By taking the limit for $\Delta t\to0$ of, say, $P_{B}$ we have

$$\lim_{\Delta t\to0} P_{B}=\frac{\frac{u(o_{B})}{1-e^{-\frac{2u(o_{B})b}{D}}}}{\frac{u(o_{B})}{1-e^{-\frac{2u(o_{B})b}{D}}}+\frac{u(o_{A})}{1-e^{-\frac{2u(o_{A})\left|a\right|}{D}}}}$$

Again, setting $\left|a\right|=\frac{1}{p}$ and $b=\frac{1}{1-p}$, the inverse of lottery *L_1_*’s probabilities , we recover the expression previously derived in , showing the equivalence between the continuous and discrete representations.

Let us therefore switch to an equivalent discrete version of Fig 2 in the manuscript, shown in S2 Fig. The random walk starts in the center of the starfish (black point) and can be absorbed in any of the absorbing states located at the end of each branch. Note that the transition probabilities are different for each segment, in order to correctly represent the different outcome-dependent potentials, while the distance of the absorbing states from the center is different for each segment, to represent the different lottery probabilities. Recall that the probability of choosing $L_{1}$ (resp. $L_{2}$), denoted by $P_{w}(L_{1})$ (resp. $P_{w}(L_{2})$), is given by the probability to be absorbed either along branch $1_{a}$ or $1_{b}$(resp.$2_{c}$ or $2_{d}$)

$$P_{w}(L_{1})=P_{w}(1a)+P_{w}(1b),\text{ }P_{w}(L_{2})=P_{w}(2c)+P_{w}(2d)$$

where the subscript $w$ indicates that we are working in the discrete random walk representation. The branch absorption probabilities can be obtained again recursively. For instance, the probability of being absorbed along branch $1_{a}$ satisfies the following equation

$$\begin{matrix} P_{w}\left(1a\right)=\frac{1}{4}\left[P_{R}\left(1,n_{A},p_{A}\right)+P_{L}\left(1,n_{A},p_{A}\right)P_{w}\left(1a\right)\right]+ \\ +\frac{1}{4}\left[P_{R}\left(n_{B},1,1-p_{B}\right)+P_{R}\left(n_{C},1,1-p_{C}\right)+P_{R}\left(n_{D},1,1-p_{D}\right)\right]P_{w}\left(1a\right) \end{matrix}$$

Solving Eq. for $P_{w}\left(1a\right)$ gives

$$P_{w}\left(1a\right)=\frac{P_{R}\left(1,n_{A},p_{A}\right)}{P_{R}\left(1,n_{A},p_{A}\right)+P_{L}\left(n_{B},1,1-p_{B}\right)+P_{L}\left(n_{C},1,1-p_{C}\right)+P_{L}\left(n_{D},1,1-p_{D}\right)}$$

Similar expressions hold for the other absorption probabilities. To ensure the equivalence between continuous and discrete representation we set

$$\begin{matrix} n_{A}=\frac{a}{\Delta x}=\frac{a}{\sigma\sqrt{\Delta t}}, p_{A}=\frac{1}{2}+\frac{u(o_{A})\sqrt{\Delta t}}{2\sigma} \\ n_{B}=\frac{b}{\Delta x}=\frac{b}{\sigma\sqrt{\Delta t}}, p_{B}=\frac{1}{2}+\frac{u(o_{B})\sqrt{\Delta t}}{2\sigma} \\ n_{C}=\frac{c}{\Delta x}=\frac{c}{\sigma\sqrt{\Delta t}}, p_{C}=\frac{1}{2}+\frac{u(o_{C})\sqrt{\Delta t}}{2\sigma} \\ n_{D}=\frac{d}{\Delta x}=\frac{d}{\sigma\sqrt{\Delta t}}, p_{D}=\frac{1}{2}+\frac{u(o_{D})\sqrt{\Delta t}}{2\sigma} \end{matrix}$$

The (asymptotic) choice probability is finally retrieved by taking the continuous time limit of $P_{w}(L_{1})$, giving

$$\lim_{\Delta t\to0} P_{w}(L_{1})=\frac{\frac{u(o_{A})}{1-e^{-\frac{2u(o_{A})a}{D}}}+\frac{u(o_{B})}{1-e^{-\frac{2u(o_{B})b}{D}}}}{\frac{u(o_{A})}{1-e^{-\frac{2u(o_{A})a}{D}}}+\frac{u(o_{B})}{1-e^{-\frac{2u(o_{B})b}{D}}}+\frac{u(o_{C})}{1-e^{-\frac{2u(o_{C})c}{D}}}+\frac{u(o_{D})}{1-e^{-\frac{2u(o_{D})d}{D}}}}$$

As for the time-dependent generalization of Eq. , once the approximate solution for the one-dimensional case (Eq. ) is “accepted”, a similar recursive argument can be applied to obtain the correspondent version on the starfish graph

With

**References**

[1] Gardiner, C. (2009). *Stochastic methods* (Vol. 4). Berlin: Springer.

[2] Feller, W. (2008). *An introduction to probability theory and its applications, vol 2*. John Wiley & Sons.
